# Site-specific ^68^Ga-labeled nanobody for PET imaging of CD70 expression in preclinical tumor models

**DOI:** 10.1186/s41181-023-00194-3

**Published:** 2023-04-24

**Authors:** Jonatan Dewulf, Tal Flieswasser, Tim Delahaye, Christel Vangestel, Alan Miranda, Hans de Haard, Julie Jacobs, Evelien Smits, Tim Van den Wyngaert, Filipe Elvas

**Affiliations:** 1 Molecular Imaging Center Antwerp (MICA), Integrated Personalized and Precision Oncology Network (IPPON), Faculty of Medicine and Health Sciences, University of Antwerp, Universiteitsplein 1, 2610 Wilrijk, Belgium; 2 Center for Oncological Research (CORE), Faculty of Medicine and Health Sciences, IPPON, University of Antwerp, Universiteitsplein 1, 2610 Wilrijk, Belgium; 3 argenx BV, Industriepark 7, Zwijnaarde, 9052 Gent, Belgium; 4 Nuclear Medicine Department, Antwerp University Hospital, Drie Eikenstraat 655, 2650 Edegem, Belgium

**Keywords:** CD70, Nanobody, ImmunoPET, Tumor imaging, Cancer

## Abstract

**Background:**

CD70-CD27 is a costimulatory ligand-receptor pair in the tumor necrosis factor receptor family. With only limited expression in normal tissues, CD70 is constitutively expressed in a variety of solid tumors and hematologic malignancies, facilitating immunosuppression through CD27 signaling in the tumor microenvironment by enhanced survival of regulatory T cells, induction of T cell apoptosis, and T cell exhaustion. Consequently, CD70 is an increasingly recognized target for developing antibody-based therapies, but its expression patterns vary among different tumor types in spatial distribution, magnitude of expression and percentage of positive cells. In that regard, individual confirmation of CD70 expression at screening and during treatment could enhance the successful implementation of anti-CD70 therapies. Here, we developed a gallium-68 (^68^Ga) radiolabeled single-domain antibody-fragment targeting CD70 for in vivo positron emission tomography (PET) imaging.

**Results:**

An anti-CD70 VHH construct containing a C-direct-tag with a free thiol was developed to enable site-specific conjugation to a NOTA bifunctional chelator for ^68^Ga radiolabeling. [^68^Ga]Ga-NOTA-anti-CD70 VHH was obtained in good radiochemical yield of 30.4 ± 1.7% and high radiochemical purity (> 94%). The radiolabeled VHH showed excellent in vitro and in vivo stability. Specific binding of [^68^Ga]Ga-NOTA-anti-CD70 VHH was observed on CD70^high^ 786-O cells, showing significantly higher cell-associated activity when compared to the blocking condition (*p* < 0.0001) and CD70^low^ NCl-H1975 cells (*p* < 0.0001). PET imaging showed specific radiotracer accumulation in CD70 expressing human tumor xenografts, which was efficiently blocked by prior injection of unlabeled anti-CD70 VHH (*p* = 0.0029). In addition, radiotracer uptake in CD70^high^ tumors was significantly higher when compared with CD70^low^ tumors (*p* < 0.0001). The distribution of the radioactivity in the tumors using autoradiography was spatially matched with immunohistochemistry analysis of CD70 expression.

**Conclusion:**

[^68^Ga]Ga-NOTA-anti-CD70 VHH showed excellent in vivo targeting of CD70 in human cancer xenografts. PET imaging using this radioimmunoconjugate holds promise as a non-invasive method to identify and longitudinally follow-up patients who will benefit most from anti-CD70 therapies.

**Supplementary Information:**

The online version contains supplementary material available at 10.1186/s41181-023-00194-3.

## Background

Over the past decades, immunotherapy has made remarkable contributions in the fight against cancer, introducing a new strategy to destroy cancer cells through activation of the host immune system (Zhao et al. 2020). Despite impressive anti-tumor effects and extended patient survival, only a minority of all treated patients respond, and life-threatening toxicity can occur (Tang et al. 2021; Zou et al. 2016). Indeed, tumor cells can escape immune surveillance and develop resistance to immunotherapy. Examples of resistance mechanisms include changes in the expression of coinhibitory or costimulatory receptors in the tumor microenvironment (TME), resistance to cytokine signaling, and irreversible T-cell exhaustion (Sharma et al. 2017). Therefore, strategies to increase the proportion of patients gaining a benefit from these treatments and increasing the durability of immune-mediated tumor response are still urgently needed. In this respect development and validation of new immunotherapy biomarkers is critical.

Immune responses are commonly evaluated by measuring blood biomarkers or invasive methods (e.g., biopsies of tumor tissue, spleen and lymph nodes) that cannot provide comprehensive information of the entire tumor and metastases because of disease heterogeneity. Moreover, routine anatomical imaging using established clinical response criteria [i.e., immune-related response criteria (iRC)] can be difficult to interpret due to pseudo-progression and only provides late information about tumor size, leaving out important functional tumor information (Nishino et al. 2017). To overcome some of these issues, additional information can be obtained using whole-body molecular imaging techniques, e.g., Positron Emission Tomography (PET), with specific radiotracers to capture an earlier and more detailed picture of the tumor phenotype. PET imaging of tumor responses during immunotherapy could provide valuable insights on therapeutic effectiveness at much earlier time points than currently available, enable timely intervention in treatment selection to avoid unnecessary systemic toxicity, and offer a unique tool for the mechanistic understanding during future drug development. Given this, the development of PET radiotracers that give early indications of response to treatment is of high clinical importance and may facilitate better management of cancer patients following immunotherapies.

The CD70-CD27 axis, belonging to the tumor necrosis factor (TNF) superfamily, has become an attractive target to exploit in oncology. CD70 is only transiently expressed on antigen-activated B and T cells, subset of NK cells and mature dendritic cells under physiological conditions (Borst et al. 2005; Tesselaar et al. 2003). In contrast, CD70 is aberrantly expressed on malignant cells and facilitates immune evasion through the TME and tumor progression (Flieswasser et al. 2022). Of note, the CD70 overexpressing cancers include diseases in need of better treatment options, including renal cell carcinoma, cervix carcinoma, glioblastoma, and lung carcinoma. Interestingly, CD70 expression was found to be even higher in metastatic lung carcinoma, pancreatic carcinoma and osteosarcoma, suggesting the importance of CD70 in the progression of the disease (Flieswasser et al. 2019). With only limited expression in normal tissues, CD70 is an increasingly recognized target for developing antibody-based therapies. As such, several antibody–drug conjugates and monoclonal antibodies have been developed and are undergoing clinical evaluation in patients with advanced solid and hematological cancers (Starzer and Berghoff 2020). Phase 1 and 2 trials are ongoing with cusatuzumab, a first-in-class anti-CD70 monoclonal antibody, in acute myeloid leukemia (AML) in combination with azacitidine and/or venetoclax (NCT04150887). In addition, another antibody, SEA-CD70, has recently started a Phase 1 trial, enrolling patients with myelodysplastic syndrome or AML (NCT04227847). Next to antibodies, several CD70-targeting chimeric antigen receptor (CAR) T-cell therapies have more recently entered the clinical (trial) arena showing great success in hematological malignancies, and are currently enrolling patients with advanced/metastatic renal cell carcinoma (NCT04438083, NCT04696731), T-cell lymphoma (NCT04502446), AML, multiple myeloma and non-Hodgkin lymphoma (NCT04662294) (Starzer and Berghoff 2020). Despite holding great potential to treat both solid and hematological cancers, CD70 targeting therapies have shown two important limitations: not all patients respond and the occurrence of dose-limiting toxicity. This is due to the fact that CD70 expression patterns in patients varies among the different tumor types in spatial and temporal distribution, magnitude of expression and percentage of positive cells (Aftimos et al. 2017). Consequently, individual confirmation of CD70 expression over time is essential for the selection of patients that are most likely to benefit from anti-CD70 therapies.

Here, we developed a gallium-68 (^68^Ga) radiolabeled single-domain antibody-fragment (sdAb, also known as Nanobody or VHH) targeting CD70, exploiting the high stability, specificity, and fast clearance of sdAbs, for in vivo PET imaging of CD70 expression. The sdAb construct contains a C-Direct tag with a free thiol that enables a site-specific labeling procedure, which avoids the risk of compromising of antigen-binding capacity and provides a uniform product more amenable to clinical translation. After radiolabeling and assessing stability, the functionality of the new tracer was validated in vitro and in vivo using CD70 expressing human cell line-derived xenografts mouse models.

After this preclinical proof-of-concept, we anticipate that targeting of CD70, in addition to personalizing immunotherapy, may provide an objective quantifiable measure of response that will expedite future development of immunotherapeutic approaches targeting CD70 and optimize combinatorial therapeutic strategies.

## Methods

All reagents were obtained from Merck unless stated otherwise. All buffers used for reduction, conjugation, and radiolabeling were purified from metal contaminants using Chelex-100 before use or prepared with TraceSELECT™ water (Fluka).

### Reduction and site-specific conjugation of anti-CD70 VHH

The generation and characterization of the anti-CD70 VHH was performed as described in supplementary information. This VHH was extended with a C-terminal C-Direct tag for improved purification and to enable site-specific conjugation of the VHH to an unpaired cysteine using maleimide chemistry as described before (Heukers et al. 2019).

The anti-CD70 VHH fragment containing a C-Direct tag (3 mg, 1 mg/mL, 107B8) was reduced by addition of 5–8 molar equivalents tris(2-carboxyethyl)phosphine (TCEP, Acros Organics) in a 0.025 M sodium borate buffer (pH 8.0) containing 0.025 M NaCl, 1 mM DTPA (total volume of 1 mL) followed by incubation for 2 h at 37 °C (Sun et al. 2005). Next, the reaction mixture was cooled down on ice and immediately followed by addition of tenfold molar excess of NOTA-maleimide (CheMatech). After incubation for 1 h at 4 °C, the conjugated VHH solution was purified from excess maleimide-NOTA and dimer unconjugated VHH via size-exclusion chromatography (SEC) (Superdex 75 Increase 10/300 GL column, Cytiva) with elution in 0.1 M NH_4_OAc (pH 7.0), followed by concentration of the conjugate using a Vivaspin2 column (cut-off 5 kDa, Sartorius). The protein concentration was measured using a spectrophotometer (Genesys 10S UV–VIS) at 280 nm (MW = 16,267 Da, Ɛ = 31,985 M^−1^ cm^−1^). The resulting conjugate was analyzed by electrospray ionization quadrupole time-of-flight (ESI-Q-ToF, Waters, Centre for Proteomics, University of Antwerp).

The equilibrium dissociation constant (K_D_) of the unmodified and NOTA-conjugated VHH was measured by surface plasmon resonance (SPR). Procedures are described in the supplementary information.

### ***Radiolabeling of anti-CD70 VHH with ***^***68***^***Ga***

The site-specific conjugated NOTA-anti-CD70 VHH fragment (100 µg) was diluted in 1 mL of 1 M NaOAc buffer (pH 4.5–4.7) and incubated with 1 mL [^68^Ga]Ga^3+^ eluate (780-870 MBq, Galli Ad, IRE ELiT) for 10 min at 50 °C. The crude radiolabeled VHH reaction mixture was purified by SEC using a PD-10 desalting column (GE Healthcare) eluted with sterile 0.01 M phosphate-buffered saline (PBS, pH 7.4). Radiochemical purity (RCP) was evaluated using radio-iTLC (0.1 M sodium citrate buffer (pH 5.0); [^68^Ga]Ga-NOTA-anti-CD70 VHH: Rf = 0; [^68^Ga]Ga^3+^: Rf = 1) and SEC as described above. The non-decay-corrected radiochemical yield was calculated based on the activity obtained after PD-10 purification. The stability assessment was performed as described in the supplementary information.

### Cell binding and affinity measurement

CD70 expression on human Burkitt's lymphoma Raji (ATCC: CCL-86), renal cell carcinoma 786-O (ATCC: CRL-1932), and lung adenocarcinoma NCl-H1975 (ATCC: CRL-5908) cell lines was evaluated using flow cytometry analysis.

The binding specificity of the radiolabeled VHH was evaluated on Raji, 786-O, and NCl-H1975 cell lines.

The binding affinity (dissociation constant, Kd) of the radiolabeled VHH was determined on CD70-positive Raji cells using a cell saturation assay. Raji cells were selected since they have a very slow rate of ligand-receptor complex internalization, whereas 786-O cells are rapidly internalizing (Additional file 1: Fig. S2) (Silence et al. 2011). All procedures are detailed in supplementary information.

### Biodistribution and metabolic stability studies

Experimental procedures and protocols were performed following European Directive 86/609/EEC Welfare and Treatment of Animals and were approved by the local ethical committee (2017–70, University of Antwerp, Belgium). All animals were housed under environmentally controlled conditions (12 h light/dark cycle, 20–24 °C and 40–70% relative humidity) in individually ventilated cages with food and water ad libitum. Animals were assigned to experimental groups using simple randomization.

Immunodeficient female nude mice (Charles River Laboratories, 5–7 weeks old, 20–25 g) were intravenously (i.v.) injected in the lateral tail vein with [^68^Ga]Ga-NOTA-anti-CD70 VHH fragment (1–4.7 µg/0.06–0.29 nmol; ~ 1.5–2.5 MBq/mouse, n = 3/time point) in 200 µL sterile saline. At different time points after radiotracer injection (15, 30, 60 and 90 min) animals were sacrificed, and the main organs and tissues were harvested, weighed, and the amount of radioactivity measured using an automated γ-counter. Uptake levels of the radiotracer were expressed as the mean percentage of the injected dose per gram (%ID/g). Metabolic stability was assessed as described in supplementary information.

### PET/CT imaging in tumor models

To generate tumor-bearing mice, immunodeficient female nude mice were subcutaneously injected in the right or left hindlimb with 786-O (4.5–8 × 10^6^, CD70^high^) or NCl-H1975 (3–5 × 10^6^, CD70^low^) cells in 100 µL sterile PBS. Tumor volume was measured twice weekly using a caliper and calculated according to the formula: 0.5 x (length x width^2^). Approximately 2–4 weeks post tumor inoculation, the tumors reached a volume around 100 mm^3^, and 786-O and NCl-H1975 xenografts were i.v. injected in the lateral tail vein with [^68^Ga]Ga-NOTA-anti-CD70 VHH (4–6.7 µg/0.25–0.41 nmol; ~ 6.4–7.4 MBq/mouse, n = 7). To assess radiotracer specificity, a subset of 786-O xenografts (n = 3) was injected i.v. with unlabeled anti-CD70 VHH fragment (20–66-fold molar excess) 1 h prior to radiotracer injection. The animals were placed on the animal bed in the scanner under isoflurane anesthesia (5% for induction, 2% for maintenance), and immediately after radiotracer injection, a dynamic PET acquisition was performed for 1 h followed by 10 min whole-body CT scan (Inveon µPET/CT scanner, Siemens). Further details are provided in the supplementary information. After image acquisition mice were sacrificed, and the main organs, tissues and tumors were harvested and weighed. The amount of radioactivity was measured using an automated γ-counter and expressed as %ID/g. Immediately after γ-counting, the tumors were processed for ex vivo analysis as described in the supplementary information.

### Statistical analysis

Data were expressed as mean ± standard deviation (SD). Flow cytometric data was analyzed using FlowJo v10.7.1 software (TreeStar Inc). Statistical analysis and graphical presentation were performed using GraphPad Prism version 9.3.1 (RRID:SCR_002798). Statistical significance between two data sets was evaluated using the two-tailed unpaired t test. Alternatively, data consisting of more than two groups were analyzed using one-way analysis of variance (ANOVA), performing Bonferroni’s post hoc test for multiple comparisons. Differences between groups were considered statistically significant if the *p* value was less than 0.05. * indicates *p* < 0.05, ** indicates *p* < 0.01, *** indicates *p* < 0.001 and **** indicates *p* < 0.0001.

## Results

### Site-specific anti-CD70 VHH fragment conjugation

In this study, we have used a site-specific labelling approach by extending the anti-CD70 VHH with a C-terminal C-Direct tag, allowing directional conjugation of the NOTA chelator to an unpaired cysteine using maleimide-thiol chemistry (Heukers et al. 2019). This strategy allowed us to obtain a single and homogeneous end product, and because the free thiol in the C-Direct tag is positioned distal of the antigen-binding loops of the VHH, to increase the possibility of maintaining the original binding integrity of the VHH. The site-specifically modified VHH was obtained in a yield of 44.2 ± 8.5% (n = 3), with a purity of > 95%, as assessed by SEC (Additional file 1: Fig. S3). The site-specific conjugation resulted in a homogeneous product of one anti-CD70 VHH bound to one NOTA chelator (Additional file 1: Fig. S4A, B).

The binding kinetics to CD70 with unconjugated and NOTA-conjugated VHHs were measured by SPR on immobilized human and mouse CD70 recombinant protein. The selected anti-CD70 VHH has been shown to be specific for human CD70 as no binding to mouse CD70 was observed. The site-specifically conjugated NOTA-anti-CD70 VHH showed a K_D_ value in the low nanomolar range in accordance with the K_D_ value of the unconjugated anti-CD70 VHH with a C-Direct tag (Table 1), which suggests that conjugation with a NOTA chelator had no impact on the functionality and affinity of the anti-CD70 VHH (see Additional file 1: Table S1 and Fig. S1 for extended data).

**Table 1:** Binding affinity kinetics (equilibrium dissociation constant (K_D_), association (k_a_) and dissociation (k_d_) rates) of anti-CD70 VHHs as determined by SPR

Ligand	Analyte	k_a_ (1/M.s)	k_d_ (1/s)	K_D_ (nM)
Anti-CD70 VHH C-Direct tag	Human CD70	1.16 × 10^6^	1.18 × 10^–3^	1.020
Anti-CD70 VHH C-Direct tag	Mouse CD70	No binding		
NOTA-anti-CD70 VHH	Human CD70	7.33 × 10^5^	1.23 × 10^–4^	0.208
Anti-CD70 VHH biotin-tag	Human CD70	8.38 × 10^5^	1.22 × 10^–4^	0.145

### *Radiolabeling and *in vitro* stability*

After site-specific conjugation with maleimide-NOTA, the VHH was radiolabeled with ^68^Ga and further characterized. Following PD-10 purification, [^68^Ga]Ga-NOTA-anti-CD70 VHH was obtained in a non-decay corrected radiochemical yield of 30.4 ± 1.7% (isolated), with an apparent molar activity of 40.6 ± 1.0 GBq/µmol and RCP > 94.3 ± 4.6% (n = 3), at the end of synthesis (Additional file 1: Fig. S5). The ^68^Ga-labeled VHH remained intact (94.9 ± 1.4%) in formulation buffer after 4 h at RT. After 3 h at 37 °C in mouse and human plasma, the radiotracer showed limited degradation, resulting in 88.6% and 90.2% intact radiotracer, respectively (Additional file 1: Fig. S6–C).

### In vitro* cell binding and affinity assay*

**Fig. 1:**
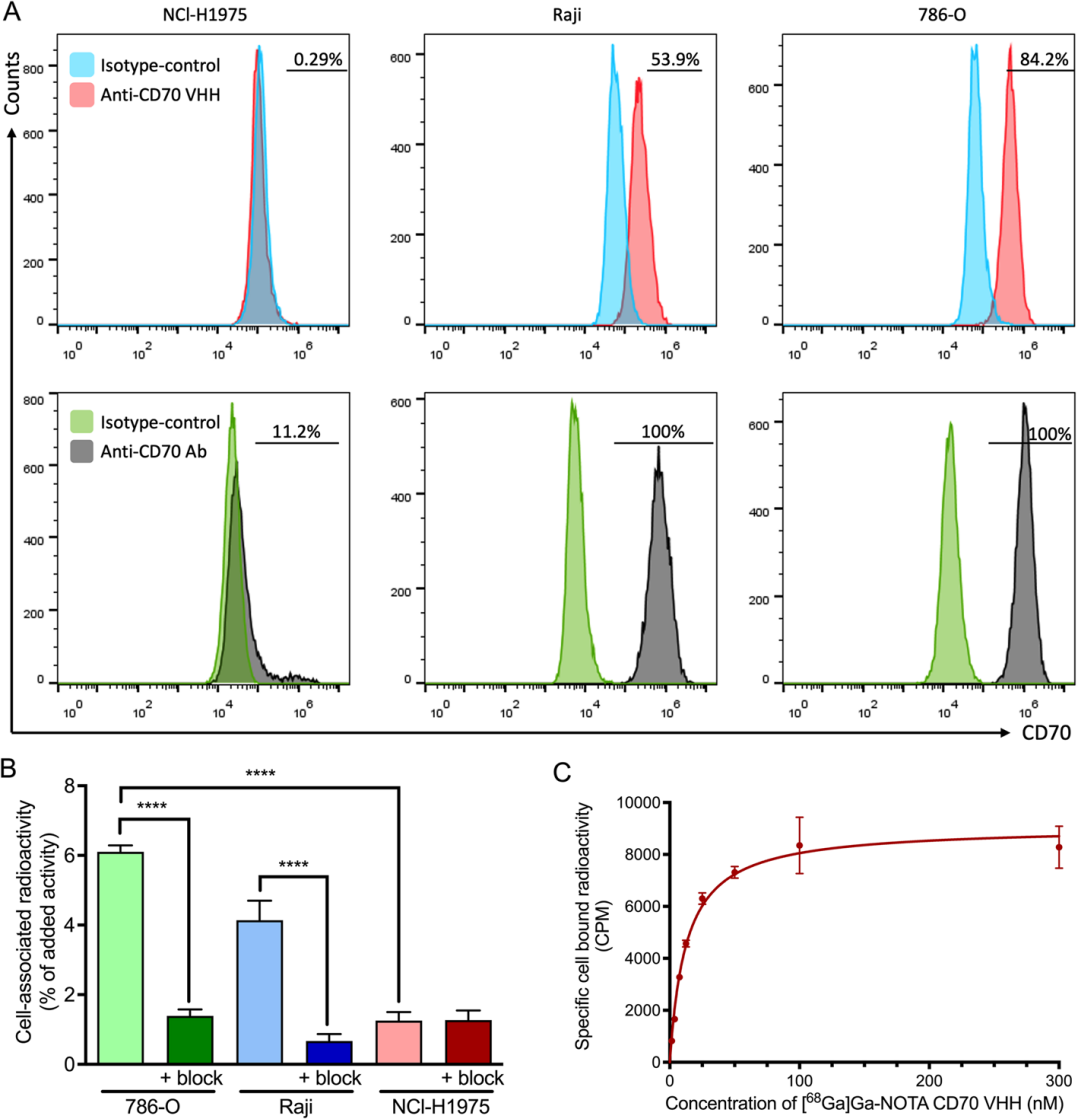
In vitro characterization of [^68^Ga]Ga-NOTA-anti-CD70 VHH. **A** Analysis of CD70 protein expression measured by flow cytometry using in the upper trace anti-CD70 VHH-Hilyte Fluor 488 (107B8) or control VHH-Hilyte Fluor 488 and in the lower trace PE mouse anti-human CD70 or PE mouse IgG3 isotype control. **B** Relative amount of cell associated activity of [^68^Ga]Ga-NOTA-anti-CD70 VHH (5 nM, 0.04 µg) on CD70^high^ and CD70^low^ cells (1 × 10^6^), or in the presence of excess unlabeled VHH (50 µg, block). (C) Specific binding of [^68^Ga]Ga-NOTA-anti-CD70 VHH on Raji cells (5 × 10^5^). (****p < 0.0001)

In order to evaluate the binding specificity of ^68^Ga-labeled VHH to CD70 in a cell assay, we have first profiled CD70 expression in different cancer cell lines by flow cytometric analysis, using either a fluorescently labeled anti-CD70 VHH (same sequence as NOTA-anti-CD70 VHH) or a PE anti-human CD70 antibody. As shown in Fig. 1A, the three cancer cell lines showed different levels of CD70 expression, with the renal cell carcinoma (RCC) cell line, 786-O, presenting the highest membrane CD70 protein levels, and the lung cancer cell line, NCl-H1975, the lowest CD70 expression. Interestingly, differences in CD70 expression level were observed between the fluorescently conjugated anti-CD70 VHH and the PE-conjugated anti-human CD70 antibody. The anti-human CD70 antibody revealed a higher percentage of CD70^+^ cells in all cell lines tested, when compared to the VHH. We hypothesized that the anti-CD70 VHH recognizes a distinct epitope on the CD70 antigen that is different from the one recognized by the mouse anti-human CD70 antibody, which represents a potential advantage for detection of this target in cancer patients treated with CD70-targeting therapies. Upon pre-incubation with the anti-CD70 therapeutical antibody ARGX-110, we continue to observe cell binding using the site-specific conjugated anti-CD70 VHH (Additional file 1: Fig. S7), confirming this hypothesis (Silence et al. 2011).

Specific binding of [^68^Ga]Ga-NOTA-anti-CD70 VHH was observed on CD70^high^ cells, with 786-O and Raji cells showing significantly higher cell-associated activity when compared to the blocking with unlabeled VHH (6.10 ± 0.19% vs. 1.39 ± 0.18%, *p* < 0.0001; and 4.14 ± 0.56% vs. 0.67 ± 0.20%, *p* < 0.0001; respectively). In addition, CD70^low^ NCl-H1975 cells showed a significantly lower amount of cell bound activity when compared to CD70^high^ cells (786-O: 6.10 ± 0.19% vs. 1.26 ± 0.18%, *p* < 0.0001; Raji: 4.14 ± 0.56% vs. 1.26 ± 0.18%, *p* < 0.0001). Importantly, the % of cell-associated activity of the radiolabeled VHH was in good agreement with the CD70 expression as assessed by flow cytometric analysis. These results confirm the specificity of the anti-CD70 VHH to its target.

The affinity of the ^68^Ga labeled VHH was determined using a cell saturation assay. The determined dissociation constant (Kd) of the [^68^Ga]Ga-NOTA-anti-CD70 VHH fragment on CD70^high^ Raji cells was 12.69 ± 1.23 nM, confirming high affinity of the VHH to the target (Fig. 1B, C).

### *Biodistribution and *in vivo* metabolic stability studies*

**Fig. 2:**
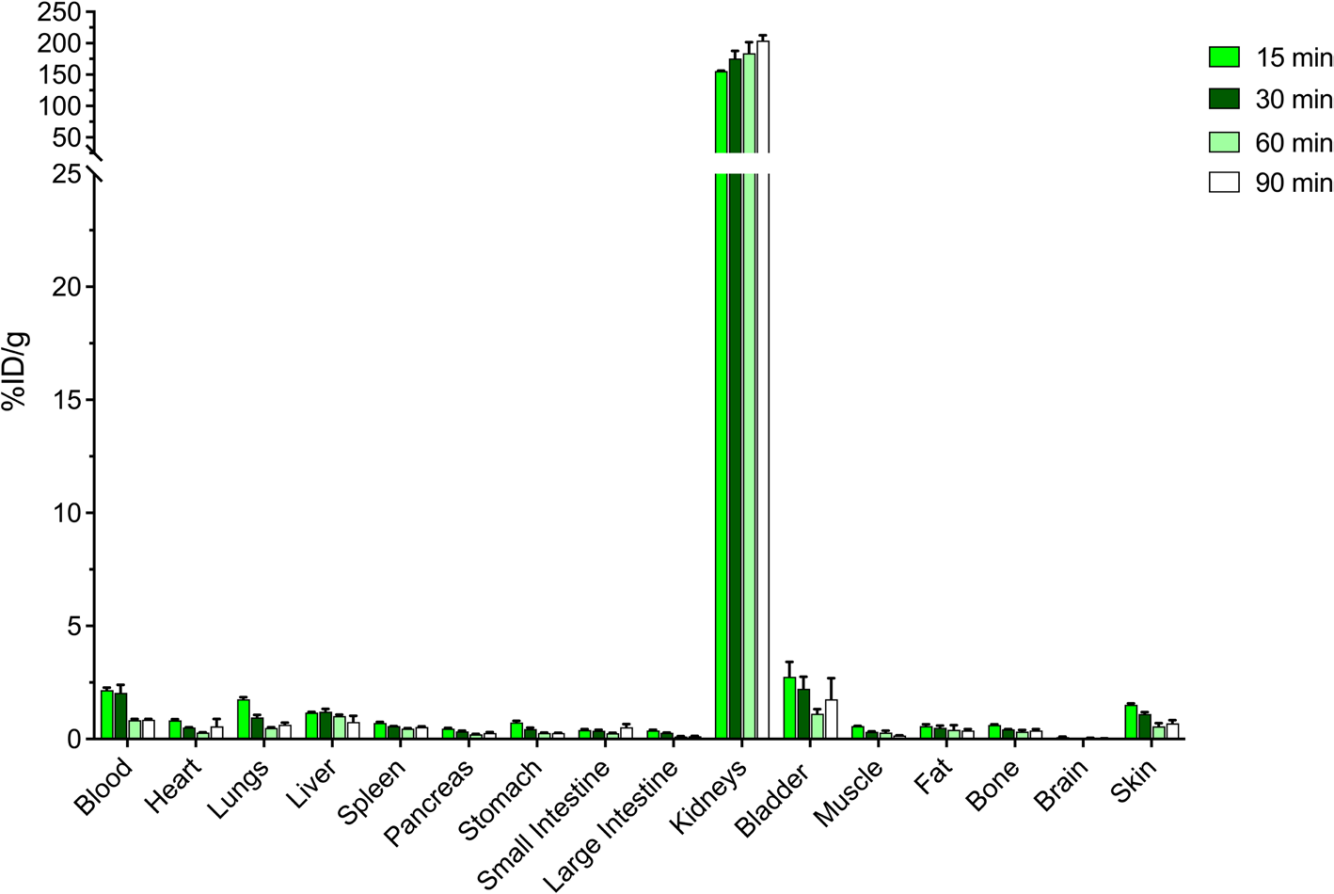
Biodistribution analysis of [^68^Ga]Ga-NOTA-anti-CD70 VHH at 15, 30, 60 and 90 min post radiotracer injection. (%ID/g = % injected dose/ gram, injected radiotracer dose = 1–4.7 µg; ~ 1.5–2.5 MBq/mouse, n = 3/time point)

Before being used for in vivo imaging of tumor models, the radiolabeled VHH should demonstrate good pharmacokinetic profile with efficient fast clearance from the blood and non-target organs and tissues. The biodistribution profile of [^68^Ga]Ga-NOTA-anti-CD70 VHH was therefore evaluated by ex vivo biodistribution. Figure 2 shows a summary of the biodistribution in normal immunodeficient mice. The ^68^Ga-labeled VHH showed fast blood clearance, with 2.17 ± 0.18% ID/g at 15 min which decreased further to 0.83 ± 0.09% at 60 min post injection (p.i.). This fast clearance was accompanied with low uptake in all organs, which together with the fast blood clearance enables low background for tumor imaging. The highest uptake of radioactivity was observed in the kidneys due to renal excretion. This renal excretion profile has also been reported for other ^68^Ga radiolabeled VHHs (Xavier et al. 2013).

[^68^Ga]Ga-NOTA-anti-CD70 VHH remained > 60% intact in plasma up to 30 min p.i. At later time points, radiotracer activity in the plasma was lower reaching 40 ± 4% at 90 min p.i. (Additional file 1: Fig. S8).

### PET/CT imaging in tumor models

**Fig. 3:**
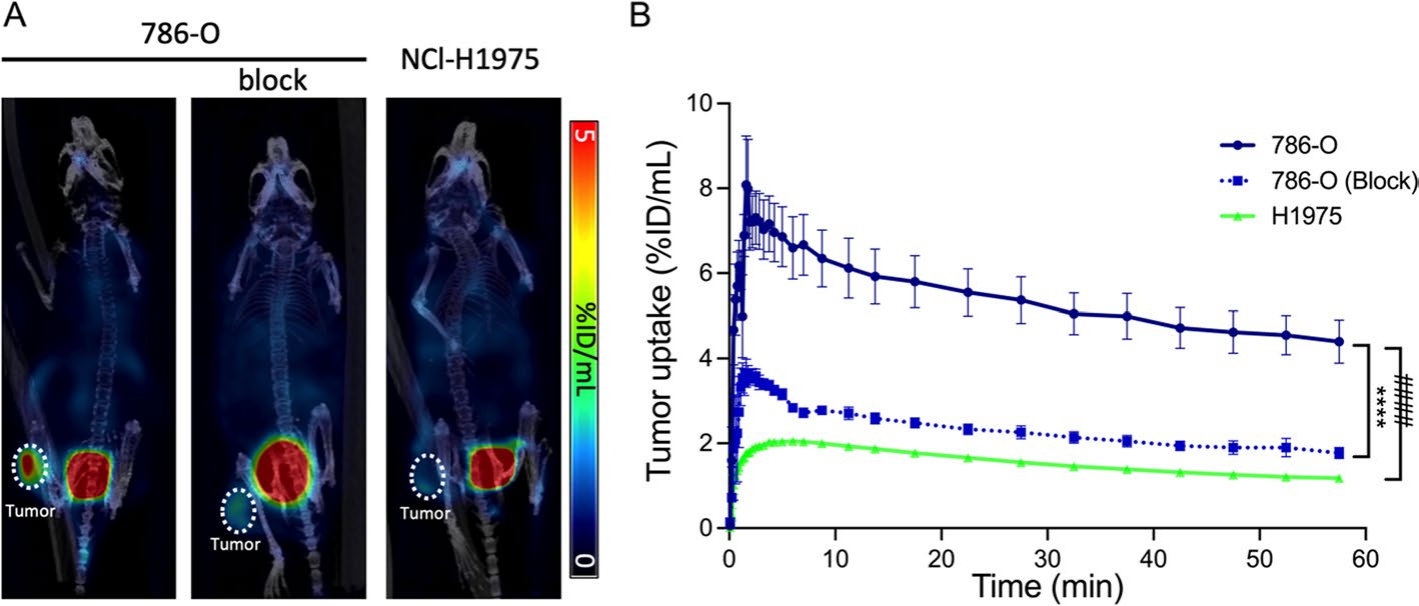
PET/CT imaging using [^68^Ga]Ga-NOTA-anti-CD70 VHH fragment—**A** Representative coronal µPET/CT images (30–60 min post injection timeframe summed activity, slice thickness 0.78 mm) of mice bearing CD70^high^ (786-O (left panel) and with block (middle panel)) and CD70^low^ tumors (NCl-H1975, right panel). **B** Time-activity curves for radiotracer distribution in the tumors are shown. (%ID/mL = % injected dose/ mL, injected radiotracer dose = 4–6.7 µg; ~ 6.4–7.4 MBq/mouse; n = 7, data are presented as mean ± SEM. ****p < 0.0001; ^####^*p* = 0.0029)

Next, we investigated the tumor targeting of the radiotracer [^68^Ga]Ga-NOTA-anti-CD70 VHH in nude mice bearing CD70^high^ tumors (786-O), or CD70^low^ tumors (NCl-H1975) as negative control using µPET/CT imaging. PET imaging showed specific radiotracer accumulation in the CD70^high^ tumors (AUC_0-60 min_: 309.3%ID/mL x min, CI_95%_ 273.1–345.5), which was efficiently blocked (AUC_0-60 min_: 132.1%ID/mL x min, CI_95%_ 126.8–137.5; *p* = 0.0029) by pre-injection of unlabeled anti-CD70 VHH (Fig. 3). In addition, radiotracer uptake in CD70^high^ tumors was significantly higher when compared with CD70^low^ tumors (AUC_0-60 min_: 89.3%ID/mL x min, CI_95%_ 84.1–94.6; *p* < 0.0001).

To validate the imaging results, at 1 h p.i. tumors were harvested, and the radioactivity was measured in a γ-counter. The ex vivo biodistribution showed a radiotracer uptake in CD70^high^ tumors of 4.17 ± 1.31%ID/g that was significantly higher than in the blocking group (2.01 ± 0.87%ID/g; *p* = 0.0043) (Additional file 1: Fig. S9). The uptake in CD70^high^ tumors was approximately 3.4 times higher than in CD70^low^ tumors (1.22 ± 0.25%ID/g; *p* < 0.0001). The tumor-to-blood and tumor-to-muscle ratios of [^68^Ga]Ga-NOTA-anti-CD70 VHH in CD70^high^ tumors were 9.99 ± 2.83 and 18.93 ± 7.85, respectively, and were significantly higher than those in CD70^low^ tumors (T/B: 1.84 ± 0.06; *p* = 0.0076, and T/M: 3.02 ± 1.10; *p* = 0.0254).

### Ex vivo* tumor analysis*

**Fig. 4:**
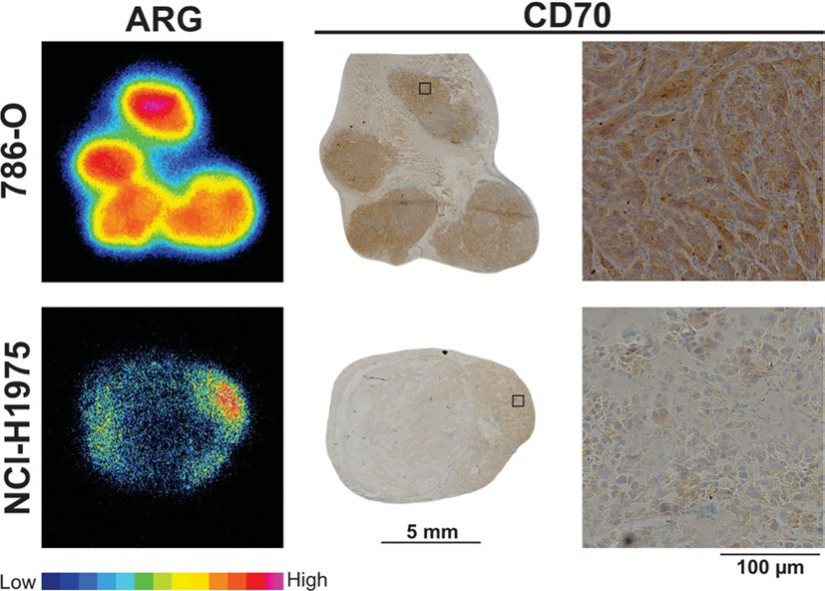
Ex vivo analysis of 786-O and NCl-H1975 tumors. Representative autoradiography (ARG) and microscopy images of adjacent histologic tumor slices stained for CD70 using a biotinylated anti-CD70 VHH

As a final step in the validation of this ^68^Ga-labeled VHH targeting CD70, we investigated the regional distribution of the radioactivity by ARG, and the expression of CD70 in the tumors by IHC. Autoradiography showed a high and homogeneous accumulation of radiotracer in CD70^high^ tumors, while tracer uptake in the autoradiographs of CD70^low^ tumors was visibly lower and restricted to only one specific tumor region at the periphery (Fig. 4). Importantly, autoradiographs showed colocalization of increased radioactivity uptake within CD70^high^ tumors areas in which there was an increased level of CD70 immunoreactivity. In agreement with [^68^Ga]Ga-NOTA-anti-CD70 VHH autoradiographs, CD70 immunoreactivity was modest in CD70^low^ tumors and was only present at the rim of the tumor. Compared with CD70^low^ tumors (11% positive area), CD70^high^ tumors showed an increased number of CD70-positive cells (91% positive area), as assessed by IHC.

## Discussion

Immunotherapy is now established as an essential weapon for treating several types of cancer, with a rapidly growing list of approved drugs and disease indications. Important examples include the immune checkpoint inhibitors mainly targeting the PD-1/PD-L1 axis, that have been used as standard of care in several cancer types. In addition, alternative pathways involved on the anti-tumor immune activity in cancer patients have been recently suggested. CD70 overexpression on tumor cells in multiple hematological malignancies and solid tumors has been therapeutically exploited preclinically and clinically as cancer-specific target (Flieswasser et al. 2022). However, expression of CD70 in the TME can be heterogeneous and therefore, evaluating CD70 expression before the start of treatment could assist in selecting patients that are more likely to benefit from anti-CD70 therapy. In addition, the possibility to longitudinally assess CD70 could help to gain further insight into the durability of anti-CD70 therapy. Therefore, to increase the proportion of patients gaining a benefit from these treatments and increasing the durability of immune-mediated tumor response, methods to improve patient selection, follow-up response to therapy and evaluate off-target effects are urgently needed. Existing companion diagnostics to select patients eligible for anti-CD70 therapy include ex vivo assessment of CD70 expression in solid and hematological tumors by immunohistochemistry and flow cytometry, respectively (Flieswasser et al. 2019; Leupin et al. 2022). However, patient biopsies are randomly sampled and often poorly reflect intra-tumoral heterogeneity, temporal target expression dynamics, and metastasis. Adding to this, tumor biopsy is an invasive method involving significant health risks and patient discomfort. Here, we describe the first radiolabeled VHH targeting human CD70 that can be used to non-invasively and longitudinally assess CD70 positivity of tumor cells.

For the development of a radiotracer for immunoPET imaging of CD70, we have decided to use a VHH fragment (also known as nanobody). In contrast to large monoclonal antibodies (150 kDa), which have been extensively explored for immunoPET imaging, nanobodies are small (12–16 kDa) and enable the generation of images with high tumor-to-background ratios shortly after administration (Dewulf et al. 2020). Importantly, the short blood half-life of nanobodies allows the use of shorter-lived isotopes, such as ^68^Ga and ^18^F, which decreases the patient radiation exposure (Rashidian and Ploegh 2020).

The anti-CD70 VHH was equipped with a C-Direct tag for site-specific conjugation of a NOTA bifunctional chelator to an unpaired cysteine using maleimide chemistry. This approach avoids potential chemical modification of amino acids involved in receptor/antigen recognition and provides a homogeneous and more consistent ^68^Ga-labeled VHH product more amenable to clinical translation. Indeed, it has been demonstrated that the unpaired cysteine in the C-Direct tag is located opposite of the complementarity determining regions (CDRs) and close to its framework, which allows functionalization of the VHH without affecting its binding characteristics (Heukers et al. 2019). Multiple site-specific methods to modify nanobodies have been described, relying on enzymatic methods, click chemistry or selective incorporation of an unpaired cysteine residue (Adumeau et al. 2016a, b). Here, the incorporation of a C-terminal unpaired cysteine allowed for convenient production, purification, and conjugation to a NOTA chelator for radiolabeling. For the reduction reaction attempts to use 2-mercaptoethylamine (2-MEA) as reducing agent, resulted in complete degradation of the VHH. Therefore, an alternative reduction protocol using TCEP was selected in order to preserve the VHH integrity. Unlike other thiol-containing reducing agents, TCEP does not have to be removed before the thiol-maleimide NOTA conjugation (Sun et al. 2005).

The resulting radiotracer, [^68^Ga]Ga-NOTA-anti-CD70 VHH, was obtained in high radiochemical yield, excellent radiochemical purity, and high apparent molar activity, which is in line with previous reports of site-specific labeled VHHs via an unpaired cysteine (Chigoho et al. 2021). The ^68^Ga-labeled VHH showed high in vitro stability both in injection buffer up to 4 h postproduction (> 94% intact), and in mouse and human plasma at 37 °C over 3 h (> 90% intact), with only the parent radiotracer being detected by radio-SEC, without signs of degradation. Additionally, stability of the radiotracer was evaluated in vivo following i.v. injection of the radiotracer in immunodeficient mice. In contrast to the results obtained in vitro, radio-TLC analysis revealed less favorable in vivo stability of [^68^Ga]Ga-NOTA-anti-CD70 VHH, with an increasing release of the radioactive metal from the radiotracer complex over time. In vivo, stability was only assessed using iTLC and can therefore only report on the radiometal complex stability and not on nanobody integrity, for this additional radio-SEC evaluation is necessary. Nevertheless, tumor-to-background contrast was not impaired by residual free Ga^3+^ activity in the blood, nor did we see increased accumulation in bone (a target for accumulation of free gallium). The reversibility of maleimide-thiol conjugation reaction has been described as a potential degradation mechanism under physiological reducing conditions (e.g., in the presence of excess glutathione). However, it is not likely that this would affect radiotracer stability only one hour after injection since this reaction generally occurs very slowly (after several days) (Baldwin and Kiick 2011). Alternatively, degradation in the C-terminal tag region could have occurred in vivo. It is known that specific sites of C-terminal tags are prone to proteolytic cleavage, which can therefore lead to the partial degradation of the radiolabeled VHH (Lykkemark et al. 2014). Yet, this remains to be experimentally investigated.

The binding ability of the site-specifically labeled VHH was determined in tumor models expressing CD70. The ^68^Ga-labeled anti-CD70 VHH was able to specifically bind to CD70-expressing cells with good affinity as seen by the low nanomolar apparent binding affinity value. Binding affinity values were in a similar range as reported for other VHH based radiotracers (Vaneycken et al. 2011). In a preliminary assay a side-by-side comparison showed a decrease of affinity when using the randomly labelled conjugate, demonstrating that this the site-specific labeling strategy provides good binding characteristics, since the attached chelator does not interfere with the CDRs recognition of the antigen (Additional file 1: Fig. S7). Conversely, other groups have encountered similar antigen targeting capacities for the site-specific and random (primary amines of lysine residues) labeled VHHs (Bridoux et al. 2020). For their clinical leads, it was shown that the loss of VHH binding affinity, by labeling of lysine residues, can be avoided by selecting VHHs without lysine residues in its antigen-binding region (Massa et al. 2014).

We have also shown that [^68^Ga]Ga-NOTA-anti-CD70 VHH was able to specifically image CD70-positive tumors with high contrast at early time points after injection. The radiotracer uptake in the tumor was comparable to previously described site-specifically labeled VHHs: 4.17 ± 1.31%ID/g vs. 1.86 ± 0.67%ID/g for the ^68^Ga-labeled targeting hPD-L1 80 min p.i., and 4.43 ± 1.50%ID/g for ^111^In-labeled targeting hHER2 90 min p.i. (Chigoho et al. 2021; Massa et al. 2014). Besides the significant tumor uptake in 786-O xenografts (CD70^high^), radiotracer retention in the kidney and bladder were observed, reflecting the typical renal excretion pattern of VHHs. The high renal uptake may hamper the low-level background activity required to detect low levels of CD70 expression expected in some cancer patients. Additionally, high accumulation of the ^68^Ga-labeled VHH in the kidneys precludes the use of this tracer for potential radionuclide therapy. Nevertheless, the high modularity of VHHs allows for easy adjustment of their binding properties and biodistribution profile. For example, it has been demonstrated that the addition of albumin-binding domains could represent an efficient strategy to decrease renal uptake, decreasing potential nephrotoxicity of VHHs equipped with therapeutic radionuclides (Lith et al. 2022). Furthermore, co-injection of lysine of gelofusine also has been reported as effective in decreasing kidney retention of VHHs (Gainkam et al. 2011).

Compared to conventional antibodies, VHHs are small and have the ability to bind antigens on hidden or unusual antigen epitopes. Using competition studies, we found that our anti-CD70 nanobody recognizes a distinct epitope on the CD70 antigen that is different from the one recognized by the CD70-blocking IgG1 monoclonal antibody, ARGX-110 (data not shown). This overcomes the limitation of interference of ARGX-110 with CD70 detection by commercially available anti-CD70 antibodies (Leupin et al. 2022). Therefore, besides the potential as an imaging tool to assess CD70 expression at the start of therapy, [^68^Ga]Ga-NOTA-anti-CD70 VHH PET imaging can be repeated over time to follow-up CD70 status during the course of treatment, thereby aiding decisions to continue or stop CD70 blockade therapy, or to add it to an ongoing treatment. Nonetheless, further studies are required to validate that expression of CD70 as assessed by PET imaging serves to predict anti-CD70 therapy response.

## Conclusion

In this study, we developed an immunoPET tracer for molecular imaging of tumor CD70 expression. We have used a site-specific radiolabeling strategy to obtain the ^68^Ga-labeled radiotracer in good radiochemical yield, favorable stability and high affinity to human CD70. Furthermore, the radiotracer was characterized by fast clearance in vivo and specific uptake in tumor xenografts. Taken together, these findings indicate the potential usefulness of [^68^Ga]Ga-NOTA-anti-CD70 VHH for individual confirmation of CD70 expression at screening and during treatment with anti-CD70 therapies, which continues to grow in importance for patient selection and follow-up in clinical trials.

## Supplementary Information

Additional file 1. Supplementary information on the generation of the anti-CD70 VHH, surface plasmon resonance measurements, flow cytometry, stability, cell binding and affinity evaluation, PET/CT imaging and ex vivo tumor analysis. Additional experimental data is described.

## Data Availability

All data generated or analyzed during this study are included in this published article and its supplementary information file.

## Abbreviations

2-MEA: 2-Mercaptoethylamine
^68^Ga: Gallium-68
AML: Acute myeloid leukemia
CAR: Chimeric antigen receptor
ESI-Q-ToF-MS: Electrospray ionization quadrupole time-of-flight mass spectrometry
HPLC: High performance liquid chromatography
iTLC: Instant thin layer chromatography
k_a_: Association constant
k_d_: Dissociation constant
K_D_: Equilibrium dissociation constant
NK cells: Natural Killer cells
NOTA: 2,2′,2″-(1,4,7-Triazacyclononane-1,4,7-triyl)triacetic acid
PET: Positron emission tomography
sdAb: Single-domain antibody-fragment
SEC: Size-exclusion chromatography
SPR: Surface plasmon resonance
TCEP: Tris(2-carboxyethyl)phosphine
TME: Tumor microenvironment
TNF: Tumor necrosis factor
