# Supplementary material for: Site-specific 68Ga-labeled nanobody for PET imaging of CD70 expression in preclinical tumor models
Source: EJNMMI Radiopharm Chem. 2023 Apr 24;8:8. doi: 10.1186/s41181-023-00194-3 (PMC10126183; doi:10.1186/s41181-023-00194-3)
Supplement: Supplementary file 1 — Additional file 1. Supplementary information on the generation of the anti-CD70 VHH, surface plasmon resonance measurements, flow cytometry, stability, cell binding and affinity evaluation, PET/CT imaging and ex vivo tumor analysis. Additional experimental data is described. [file 41181_2023_194_MOESM1_ESM.docx]

**Supplementary information**

Site-specific ^68^Ga-labeled nanobody for PET imaging of CD70 expression in preclinical tumor models.

**Authors**

Jonatan Dewulf^1^, Tal Flieswasser^2^, Tim Delahaye^3^, Christel Vangestel^1,4^, Alan Miranda^1^, Hans de Haard^3^, Julie Jacobs^3^, Evelien Smits^2^, Tim Van den Wyngaert^1,4^, Filipe Elvas^1^

[1] Molecular Imaging Center Antwerp (MICA), Faculty of Medicine and Health Sciences, University of Antwerp, Universiteitsplein 1, 2610 Wilrijk, Belgium.

[2] Center for Oncological Research (CORE), Integrated Personalized and Precision Oncology Network (IPPON), University of Antwerp, Universiteitsplein 1, 2610 Wilrijk, Belgium.

[3] argenx BV, Industriepark 7, Zwijnaarde, 9052 Gent, Belgium.

[4] Nuclear Medicine, Antwerp University Hospital, Drie Eikenstraat 655, 2650 Edegem, Belgium.

**Corresponding Author:**

Filipe Elvas, Ph.D., [filipe.elvas@uantwerpen.be](mailto:filipe.elvas@uantwerpen.be)

**Cells and culturing conditions**

Raji (ATCC CCL-86, RRID:CVCL_0511), 786-O (ATCC CRL-1932, RRID:CVCL_1051), NCI-H1975 (ATCC CRL-5908, RRID:CVCL_1511), Hut78 (ATCC TIB-161, RRID:CVCL_0337), THP-1 (ATCC TIB-202, RRID:CVCL_0006) and HL-60 (ATCC CCL-240, RRID:CVCL_0002) cells were obtained from ATCC and cultured as recommended by manufacturer instructions. Cells were incubated at 37°C in humidified conditions with 5% CO_2_ and cultured as monolayers. Adherent cells were harvested using 0.05% trypsin/EDTA solution (Invitrogen). All cell cultures were confirmed as Mycoplasma free using the Mycoalert^®^ Mycoplasma detection kit (Lonza, LT07-218).

**Generation of phage-display library and selection of anti-CD70 VHH**

Two llamas were immunized 6 times at weekly intervals with 100 µg recombinant CD70 protein (trimeric CD70, produced as described in Wyzgol et al.(1)), in the first 2 immunizations, and 50 µg protein in the next 4 immunizations(1). Afterwards, whole blood was drawn, processed and an anti-CD70 VHH phage-display library generated according to methods published before(2, 3). Briefly, from the blood of the immunized animals, peripheral blood mononuclear cells (PBMCs) were isolated using Pancoll Lymphocyte Separating Medium (Pan-Biotech; Cat N° P04-60125), RNA was isolated using the RNeasy maxi kit (Qiagen, Cat N° 75162) and RNase-free DNAse set (Qiagen, Cat N° 79254), and total cDNA was generated applying the SuperScript^TM^ III First-Strand Synthesis System (Invitrogen, Cat. N° 18080-051). This was used as a template for 2-step PCR. First, non-tagged primers were used directly on the cDNA from the llama PBMCs. The PCR product was purified and used secondly with tagged primers that will allow digestion and cloning into pre-digested vectors suited for phage display selections (phage vector). Next, the phage selections were performed on coated recombinant CD70 (5 and 0.5 µg/mL) for 3 consecutive selection rounds(4). VHH-containing periplasmic fraction of TG1 E. coli was generated for the selected clones and screened for binding to recombinant CD70 in ELISA and on CD70^+^ cells (data not shown) (4). The clones showing the highest binding to CD70 (with and without prebound ARGX-110) were selected to be produced as purified VHH fragments (3x Flag-tag) from 400 mL scale TH1 E. coli cultures, followed by purification of the periplasmic VHH using TALON^®^ Superflow™ histidine-tagged protein purification resin (Cytiva, Cat N° 28957502). The VHHs were buffer exchanged to phosphate buffered saline (PBS) and stored at 4°C.

The purified generated anti-CD70 VHHs were applied on Hut78 cells (T cell Lymphoma, Sezary Syndrome, CD70-positive cell line) in a dilution series in PBS containing 0.05% FCS. After incubation of 1 hour at 4°C, the cells were washed and incubated with a secondary detection antibody (Myc-tag (9B11) mouse mAb Alexa^®^ 647 (conjugate), Cell Signaling Technology, Cat N° 11/2016) in a 1/100 dilution for 1 hour at 4°C, detecting the myc-tag present at the purified VHH fragments. After washing the cells twice with 200 µL PBS + 0.05% FCS, the cells were resuspended in 150 µL PBS + 0.05% FCS and analyzed on a flow cytometer (FacsCanto, BD Biosciences). The data showed clear binding of the selected anti-CD70 VHH to CD70 on Hut78 cells (not shown).

**Production and purification of selected anti-CD70 VHH, and expression of a VHH construct with an unpaired cysteine**

The selected anti-CD70 VHH protein was further optimized and produced for site-directed labeling at QVQ (Utrecht, The Netherlands). The pYQVQ11 vector was used for VHH production in yeast, introducing a C-Direct tag (consisting of an unpaired cysteine, a FLAG-tag derived peptide and a C-terminal EPEA (Glu, Pro, Glu, Ala) purification tag. To improve production yields and facilitate purification from supernatant, C-Direct-tagged VHH were produced in S. cerevisiae strain VWK18, as described previously, with yields of 25-30 mg/L (n = 13)(5, 6, 7, 8). VHHs were purified from the yeast supernatant using an Äkta Start (GE Healthcare), a Capture-Select C-tag XL affinity chromatography column (Thermo Fisher Scientific) and size-exclusion chromatography (Thermo Fisher Scientific) according to the manufacturer’s protocols. Afterwards, obtained VHH was filter sterilized and stored in PBS (phosphate-buffered saline).

**Surface plasmon resonance (SPR)**

Measurements were performed on a Biacore 8K+ device (Cytiva, GE Healthcare) at 25°C and using HEPES-buffered saline (HBS; 0.01 M HEPES pH 7.4, 0.15 M NaCl, 3 mM EDTA, 0.005% Tween 20) as running buffer. The recombinant human and mouse CD70 proteins (1) were dissolved to 5 μg/mL and 2 µg/mL, respectively, in 10 mM NaOAc pH 5.0 for immobilization on a Series S CM5 sensor chip using linkage chemistry with 1-(3-(dimethylamino)propyl)-3-ethylcarbodiimide (EDC, Cytiva) and N-hydroxy-succinimide (NHS, Cytiva). Unreacted EDC-NHS linkers were blocked with 1 M ethanolamine-HCl (Cytiva). The following VHHs were tested for affinity kinetics on immobilized human and mouse CD70 protein in SPR: anti-CD70 VHH C-direct tag, NOTA-anti-CD70 VHH and anti-CD70 VHH biotin-tag. To this end, 6 different VHH dilutions were allowed to bind to the target protein for 25 sec and dissociation was monitored for 600 sec. The chip surface was regenerated by 4 consecutive injections of 10 mM Glycine-HCl pH 3.0 (Cytiva), each injection having a contact time of 15 sec at a flow rate of 30 µL/min. The equilibrium dissociation constant K_D_ was calculated by fitting the obtained sensorgrams to theoretical curves, assuming 1-to-1 binding geometries, using Biacore Insight Evaluation Software (version 4.0.8.19879) (Figure S1). The highest concentration (500 nM) for the 3 ligands did not provide a good fit and was therefore locally excluded from our analysis. For the analysis of the VHH C-direct tag, also the sensorgram at 125 nM was excluded from the fit (Table S1).

**Table S1** Global overview of the data and the quality assessment of the fitted parameters

| **Immobilized human CD70 (RU)** | **Analyte** | **Uniqueness (U-value)^a^** | **T(*k*_on_)^b^** | **T(*k*_off_)^b^** | **Concentration range for fitting** |
| --- | --- | --- | --- | --- | --- |
| 303.3 | VHH C-Direct tag | 2 | 157 | 194 | 250 – 15.6 nM |
| 363.3 | VHH-NOTA | 7 | 3350 | 3040 | 250 – 15.6 nM |
| 376.5 | VHH-biotin | 9 | 1420 | 586 | 250 – 15.6 nM |

^a^Global parameter for the quality of the fit, ^b^T-value Quality indicator for each parameter

**Flow cytometry**

For flow cytometry analysis, cells were plated overnight at 1 x 10^6^ cells/mL (24-well plate) at 37°C. The next day, cells were harvested, washed once with 0.01 M PBS and subsequently twice with ice-cold staining buffer (PBS, 2% FBS, 0.1% sodium azide). After the final wash, cells were resuspended in 100 µL staining buffer containing either anti-CD70 VHH-Hilyte Fluor 488 (107B8, argenx), control VHH-Hilyte Fluor 488 (argenx), PE mouse anti-human CD70 (BD Biosciences, Cat# 555835, RRID:AB_396158) or PE mouse IgG3 isotype control (BD Biosciences, Cat# 556659), and incubated for 30 min on ice protected from light. After incubation, cells were washed with sorting buffer (PBS, 2.5 mM EDTA, 25 mM HEPES pH 7.0, 1% FBS) and CD70 expression was analyzed on a CytoFLEX flow cytometer (Beckman Coulter).

Anti-CD70 VHH-Hilyte Fluor 488 and control VHH-Hilyte Fluor 488 were site-directionally conjugated to fluor 488 using a thiol-maleimide reaction, via a similar approach as the radiotracer. First, the VHHs were incubated with a 2.75-fold molar excess of TCEP (VWR International, Radnor, PA, USA) to reduce the C-terminal cysteine. Next, the partially reduced VHH were conjugated to HiLyteFluor 488 C2 maleimide (AnaSpec Inc, Fremont, CA, USA) according to the manufacturers protocol. Free label was removed by size-exclusion chromatography using two consequent Zeba Desalting Columns (Thermo Fisher Scientific). Degree of conjugation was determined using the Multiskan Go spectrophotometer (Thermo Fisher Scientific) and the amount of free dye was determined after size separation by SDS-PAGE (Bio-Rad) on a D-Digit fluorescence scanner (Li-COR Biosciences).

**Stability evaluation**

The stability of the ^68^Ga-labeled VHH in vitro was evaluated at room temperature over 4 h, and in mouse (Genetex) and human (Innovative research) plasma at 37°C for 3 h. At different time points, the aliquots were diluted in 0.1 M citrate buffer containing 0.1 % Tween-20 and filtered through a 0.22 µm filter before radio-SEC analysis (Figure S6).

In short, to assess metabolic stability, blood was collected via cardiac puncture in EDTA-coated tubes, and plasma fractions were obtained by centrifugation at 4,500 *g* for 7 min. The plasma fraction was spotted on a SG-TLC strip and the in vivo radiotracer integrity was analyzed by radio-iTLC as described in the main manuscript (Figure S8).

**Cell binding and affinity assays**

The binding specificity of the radiolabeled VHH was evaluated on Raji, 786-O, and NCl-H1975 cell lines. After plating overnight, 10^6^ cells were incubated with [^68^Ga]Ga-NOTA-anti-CD70 VHH at a final concentration of 5 nM (0.04 µg) in cell medium. Nonspecific binding was assessed by preincubation for 3 h at 37°C of excess of unlabeled anti-CD70 VHH fragment (50 µg). After 3 h incubation at 37°C, cells were washed once with ice-cold PBS, and the cell bound activity was measured on an automatic gamma counter (Wizard 2480, Perkin Elmer) and expressed as percentage of added activity. All measurements were performed in triplicate.

The binding affinity (dissociation constant, Kd) of the radiolabeled VHH was determined on CD70-positive Raji cells. Briefly, 5 x 10^5^ cells were allowed to equilibrate for 1 h on ice prior to the start of the experiment. After removal of the supernatant, the radiotracer solution was incubated at different concentrations (1.5 – 300 nM) for 3 h at 4°C. To assess the nonspecific binding, a subset of cells was pre-incubated with 50-fold molar excess of unlabeled anti-CD70 VHH fragment. The Kd value was calculated using GraphPad Prism version 9.3.1 (RRID:SCR_002798), with the function – “Binding, specific saturation binding”. Experiments were performed in triplicate.

The percentage of internalization of [^68^Ga]Ga-NOTA-anti-CD70 VHH was determined on the 786-O and Raji cells in non-adherent culture conditions. Briefly, to 1 × 10^6^ cells was added 1 µg nanobody solution (in 30 µL) followed by incubation for 1 h at 4°C. Next, the unbound radiotracer was collected and the cells were incubated at 37°C during different incubation times: 10, 30, 50 and 70 min. Then, the supernatants and pellets were collected, and the membrane-bound fraction was obtained by washing the pellet with a cold glycine/NaCl (pH 2.8) solution. All fractions were measured using a gamma-counter. The percentage of internalized activity was calculated as a percentage of the total cell-associated activity.

**PET/CT imaging**

PET data was reconstructed with proprietary 3D list-mode ordered subsets reconstruction with spatially variant resolution modelling using a point spread function measured for ^68^Ga in water equivalent material(9). Attenuation correction was performed using an attenuation map calculated from the CT image. Volumes of interest (VOIs) were manually drawn on the PET/CT images using PMOD (PMOD, v3.6; PMOD Technologies, RRID:SCR_016547) to delineate the regions of interest: heart, kidney, bladder, muscle and tumor and generate time activity curves (TACs). TACs of tumors were compared via calculating the area under the curve (AUC). Biodistribution was evaluated at 1h 10 min post radiotracer injection.

***Ex vivo* tumor analysis**

Immediately after the γ-counting, the tumors were snap-frozen and embedded in optimal-cutting-temperature compound (tissue-Tek; VWR) and sectioned at 100 µm for autoradiography (ARG) and adjacent sections at 10 µm for histological analysis using a cryostat (Leica biosystems). For ARG, sections were exposed to phosphor screen plates (Fujifilm) overnight and subsequently imaged in a phosphor imager system (Typhoon FLA7000, GE Healthcare). In parallel, 10 μm frozen tumor sections were used for histological analysis of CD70 expression by immunostaining using anti-CD70 VHH with a biotin tag (0.00046 mg/mL, 107B8, argenx (as prepared via protocol described before)). The slides were post fixed with 4% paraformaldehyde, rinsed with PBS-T (phosphate buffered saline with 0.1% Tween 20), and endogenous peroxidase activity was blocked with 3% H_2_O_2_ (Merck). The slides were subsequently washed with PBS-T, followed by normal serum and biotin blocking using avidin/biotin blocking kit (Vector Laboratories). Incubation with anti-CD70 was performed overnight at room temperature. The next day, the slides were washed with PBS and incubated with extravidin-HRP (Merck), followed by DAB staining (DAKO). The nuclei of the tissue sections were counterstained using Mayer’s hematoxylin (Merck).

**References**

1. Wyzgol A, Müller N, Fick A, Munkel S, Grigoleit GU, Pfizenmaier K, et al. Trimer Stabilization, Oligomerization, and Antibody-Mediated Cell Surface Immobilization Improve the Activity of Soluble Trimers of CD27L, CD40L, 41BBL, and Glucocorticoid-Induced TNF Receptor Ligand. The Journal of Immunology. 2009;183(3):1851-61.

2. de Haard HJ, van Neer N, Reurs A, Hufton SE, Roovers RC, Henderikx P, et al. A large non-immunized human Fab fragment phage library that permits rapid isolation and kinetic analysis of high affinity antibodies. The Journal of biological chemistry. 1999;274(26):18218-30.

3. Bobkov V, van der Woning B, de Haard H. Display Technologies for Generation of Ig Single Variable Domains. Methods in molecular biology. 2018;1827:129-44.

4. van der Woning B, De Boeck G, Blanchetot C, Bobkov V, Klarenbeek A, Saunders M, et al. DNA immunization combined with scFv phage display identifies antagonistic GCGR specific antibodies and reveals new epitopes on the small extracellular loops. MAbs. 2016;8(6):1126-35.

5. Gorlani A, de Haard H, Verrips T. Expression of VHHs in Saccharomyces cerevisiae. Methods in molecular biology. 2012;911:277-86.

6. Gorlani A, Hulsik DL, Adams H, Vriend G, Hermans P, Verrips T. Antibody engineering reveals the important role of J segments in the production efficiency of llama single-domain antibodies in Saccharomyces cerevisiae. Protein Eng Des Sel. 2012;25(1):39-46.

7. Thomassen YE, Meijer W, Sierkstra L, Verrips CT. Large-scale production of VHH antibody fragments by Saccharomyces cerevisiae. Enzyme and Microbial Technology. 2002;30(3):273-8.

8. van de Laar T, Visser C, Holster M, Lopez CG, Kreuning D, Sierkstra L, et al. Increased heterologous protein production by Saccharomyces cerevisiae growing on ethanol as sole carbon source. Biotechnol Bioeng. 2007;96(3):483-94.

9. Miranda A, Bertoglio D, Glorie D, Stroobants S, Staelens S, Verhaeghe J. Validation of a spatially variant resolution model for small animal brain PET studies. Biomed Phys Eng Express. 2020;6(4):045001.

**Figures**

**Figure S1** Fitted sensorgrams from SPR analysis on coated human CD70. The VHHs were applied to the coated surface in dilution series of 6 concentrations (ranging from 500 to 15.6 nM). The dotted grey line shows sensorgrams that were locally excluded from fitting because the quality of fit was not acceptable for these conditions. A) For Anti-CD70 VHH C-Direct tag, sensorgrams at 500 and 150 nm were excluded. For B) NOTA-anti-CD70 VHH and C) anti-CD70 VHH biotin tag, the sensogram at 500 nM was excluded from fitting.


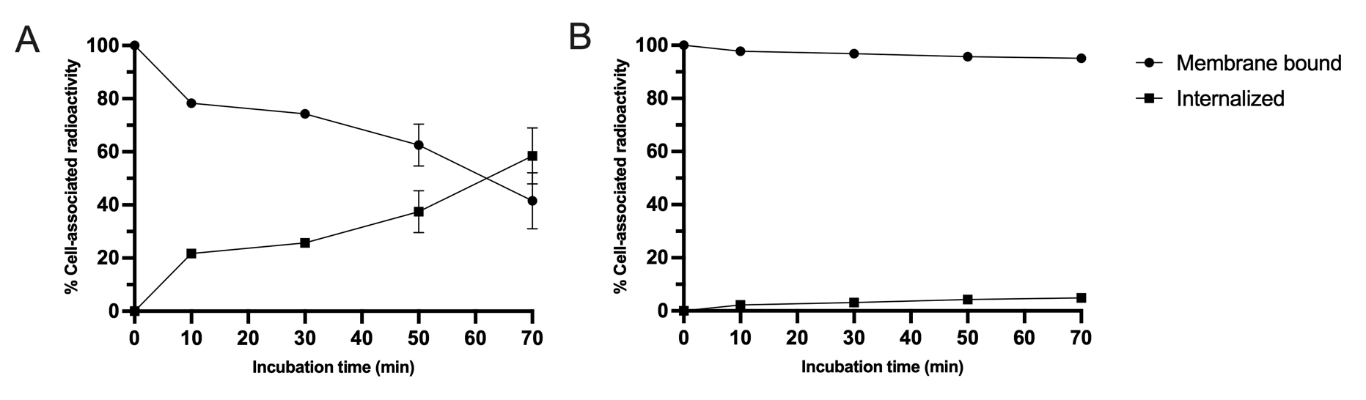


**Figure S2** Evaluation of internalization of [^68^Ga]Ga-NOTA-anti-CD70 VHH on A) 786-O and B) Raji cells. Data is expressed as a percentage of the cell-associated activity (sum of membrane bound + internalized fraction) at 0, 10-, 30-, 50- and 70-min incubation.


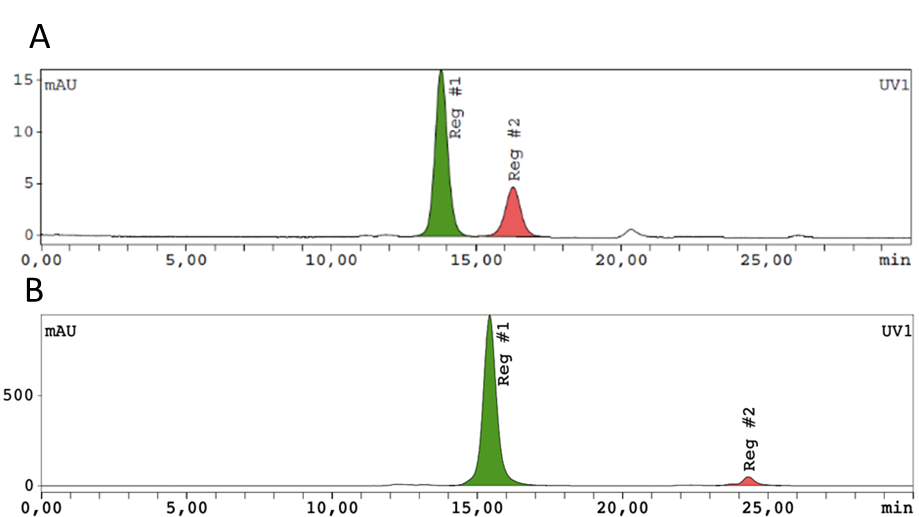


**Figure S3** SEC analysis of A) anti-CD70 VHH stock solution consisting of a dimer (reg 1) and a monomer (reg 2) peaks; and B) NOTA-conjugated anti-CD70 VHH showing >95% purity (UV-vis-chromatograms).

**Figure S4** ESI-Q-ToF mass spectrometry analysis – A) of anti-CD70 VHH monomer (16,800 Da) and B) of the site-specifically conjugated NOTA-anti-CD70 VHH (17,230 Da).


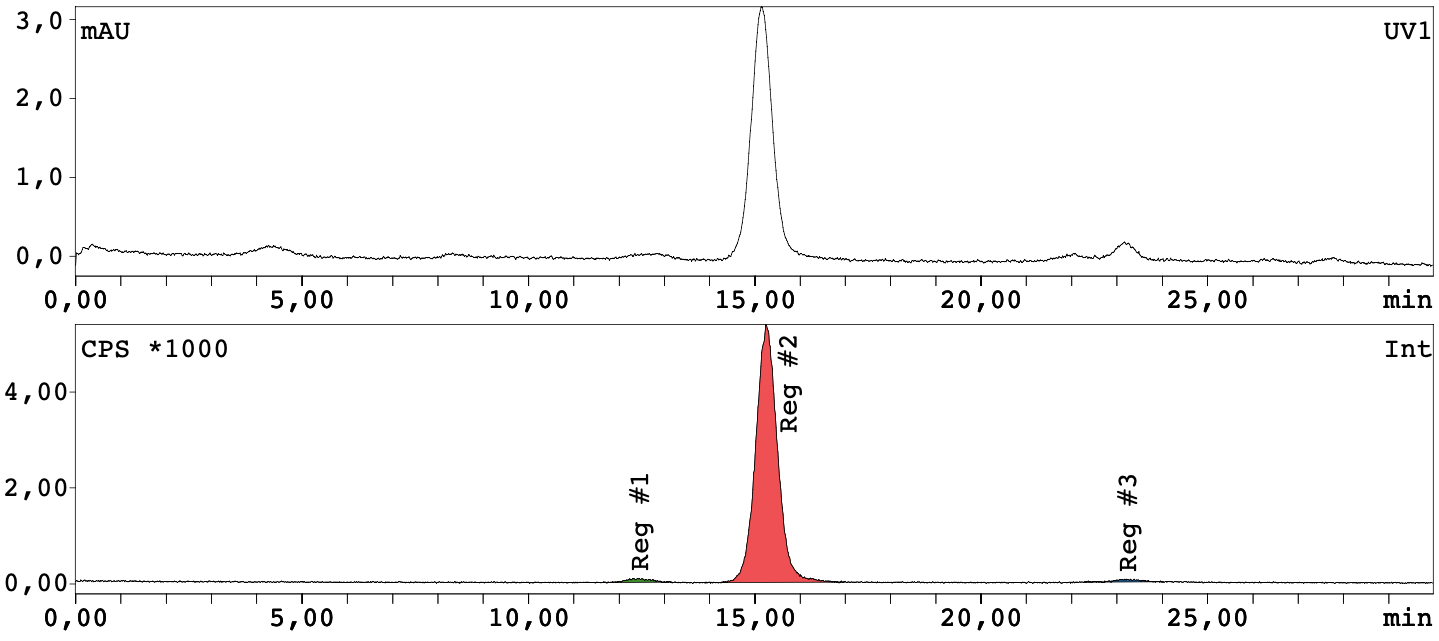


**Figure S5** SEC-HPLC chromatograms of [^68^Ga]Ga-NOTA-anti-CD70 VHH fragment. Upper trace: UV-vis-channel; Lower trace: radioactive channel.

A


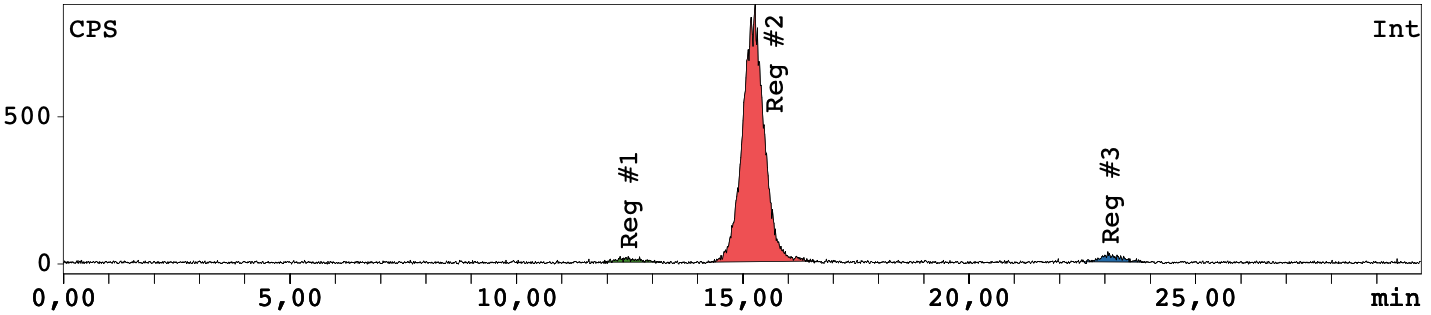


B


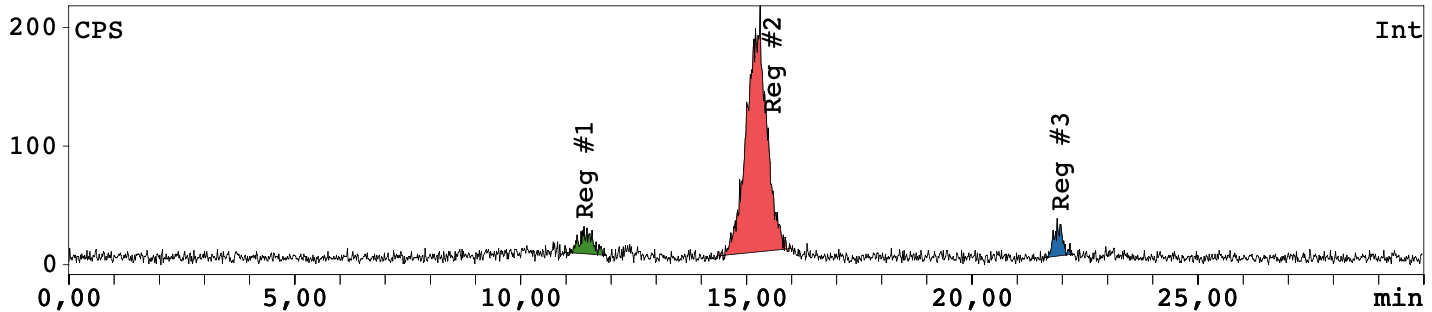


C


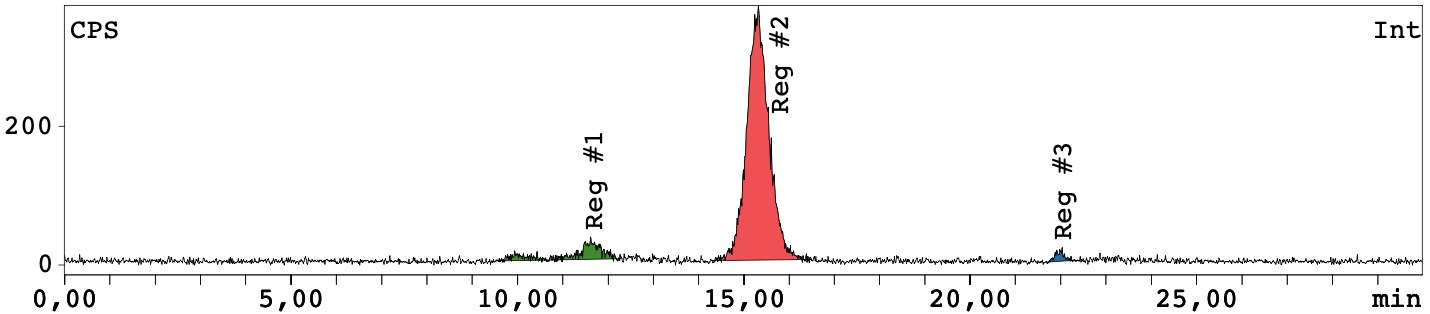


**Figure S6** *In vitro* stability assessment of [^68^Ga]Ga-NOTA-anti-CD70 VHH in A) formulation buffer at room temperature 4 h post production, and in B) mouse and C) human plasma after incubation for 3 h at 37°C.


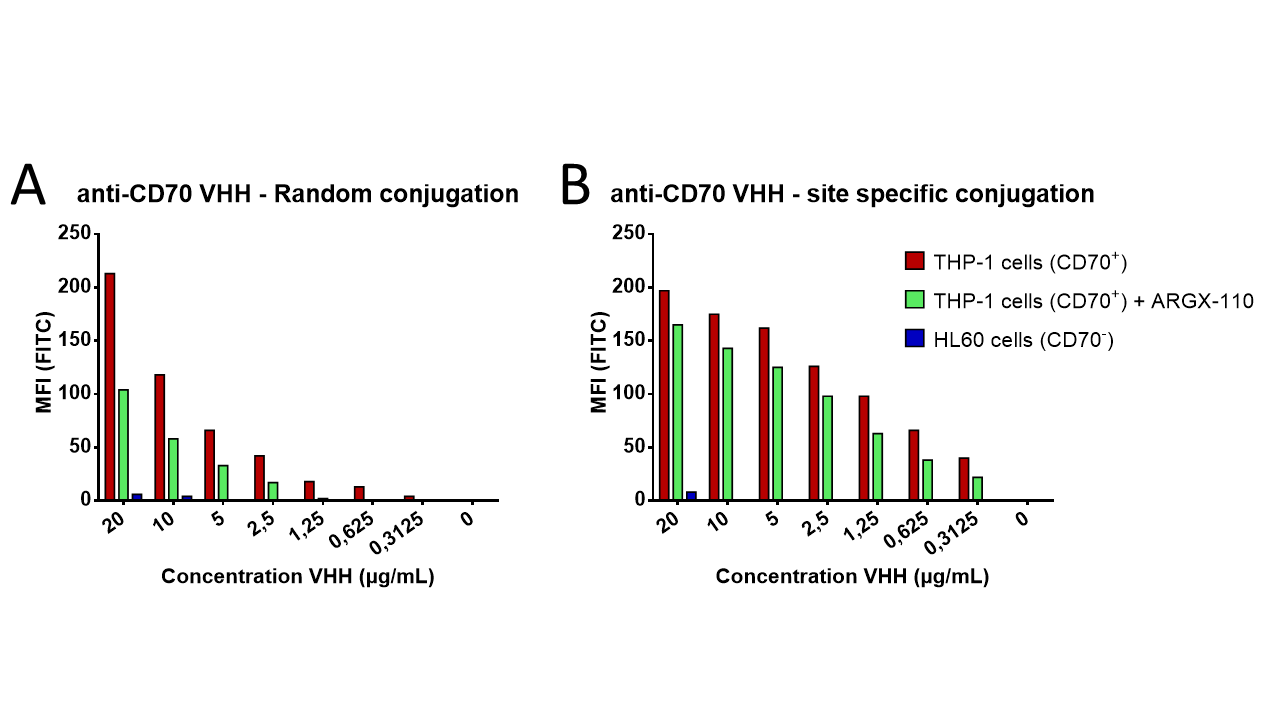


**Figure S7** Competitive preliminary binding assay for A) random and B) site specific conjugates within or without the presence of the therapeutic antibody ARGX-110 (100 µg/mL) for THP-1 (CD70+, AML) and HL60 (CD70-, AML) cells.


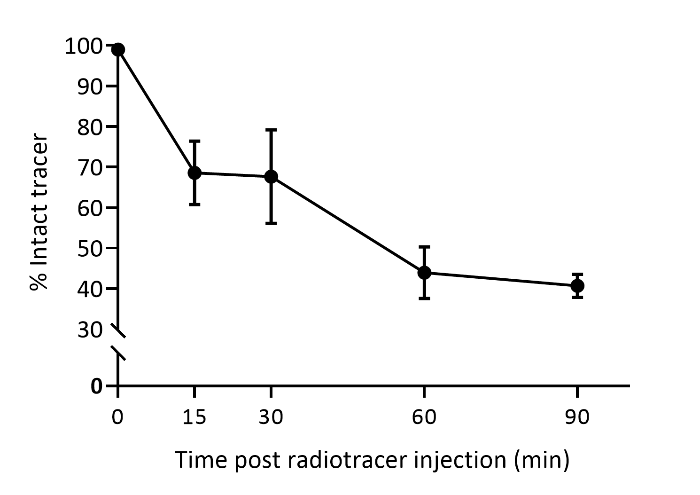


**Figure S8** *In vivo* stability study of [^68^Ga]Ga-NOTA-anti-CD70 VHH, determining metal complex stability using iTLC analysis (injected radiotracer dose = 1 – 4.7µg; ~ 1.5 – 2.5MBq/mouse, N = 3/time point).

**Figure S9** *Ex vivo* biodistribution analysis of [^68^Ga]Ga-NOTA-anti-CD70 VHH in mice bearing CD70^high^ (786-O, with and without block) and CD70^low^ tumors (NCl-H1975). (% ID/g = % injected dose/ gram, injected radiotracer dose = 4 – 6.7 µg; ~ 6.4 – 7.4 MBq/mouse; N=7, ***p* = 0.0043; *****p*<0.0001).
